# Genotypic and phylogenic analyses of cutaneous leishmaniasis in Al Ahsa, Eastern Saudi Arabia during the coronavirus disease 2019 pandemic: First cases of *Leishmania tropica* with the predominance of *Leishmania major*

**DOI:** 10.1038/s41598-022-14702-z

**Published:** 2022-06-24

**Authors:** Abdullatif S. Al-Rashed, Reem Al Jindan, Salma Al Jaroodi, Ahmed Al Mohanna, Ahmed Abdelhady, Ayman A. El-Badry

**Affiliations:** 1 Department of Microbiology, College of Medicine, Imam Abdulrahman Bin Faisal University, Dammam, Saudi Arabia; 2 Vector Borne and Diseases Prevention Center, Al Ahsa, Saudi Arabia; 3 Ministry of Health, Riyadh, Saudi Arabia

**Keywords:** Microbiology, Health care

## Abstract

During the coronavirus disease 2019 lockdown period, a surge in sandflies and cutaneous leishmaniasis (CL) cases was observed in Al-Ahsa, Saudi Arabia. Skin punch biopsies were obtained from 100 patients clinically diagnosed with CL in Al-Ahsa who had no travel history in the last 6 months. Impression smears were used following a three-step polymerase chain reaction (PCR) protocol using genus-specific primers targeting kDNA and ITS1. *Leishmania* speciation was determined by ITS1 PCR/nested PCR-restriction fragment length polymorphism and sequencing. A phylogenetic tree was constructed. The associated patient characteristics were analyzed. Using internal transcribed spacer one (ITS1)-PCR/nested PCR, 98 cases were considered true-positive CL. *Leishmania major* was the predominant species, and *Leishmania tropica* was identified in three cases. Microscopy had poor sensitivity and perfect specificity. Direct ITS1-PCR missed nine cases. Sex, residence, and treatment outcome were significantly associated with the occurrence of *Leishmania*; distribution of skin lesion(s) and treatment outcome were significantly associated with *Leishmania* genotype. This is the first time that *L. tropica* was identified as a cause of CL in human in Al-Ahsa, in addition to the predominant zoonotic species, *L. major*. We recommend using ITS1-nested PCR for negative cases by ITS1-PCR. Further exploration of *Leishmania* transmission dynamics in vectors and reservoir animals is essential for designing effective preventive measures.

## Introduction

Leishmaniasis, a vector-borne disease, is a complex disorder caused by a heterogeneous group of flagellated protozoans of one genus, *Leishmania*, and belongs to the Trypanosomatidae family that has a worldwide distribution^1,2^. Female sandfly vectors are the principal *Leishmania* transmittor^1^.

Leishmaniasis is categorized into a spectrum of diseases according to its form^1^. It is primarily classified into cutaneous leishmaniasis (CL), mucocutaneous leishmaniasis, and visceral leishmaniasis^3^. CL is considered the predominant form of leishmaniasis and is presented with one or multiple ulcerated lesions in exposed areas of the human skin^2,3^.

There are more than 20 species in the genus *Leishmania*. Species can be classified according to their geographical distribution into Old and New World leishmaniasis^2^.

Old World CL cases are caused predominantly by the zoonotic *Leishmania* (*L.*) *major* and the *L. tropica* in Pakistan, India, Iran, Palestine, Jordan, Yemen, and Saudi Arabia^4–10^; *L. major*, *L. infantum*, and *L. aethiopica* in Saharan Africa and Ethiopia^11–13^; *L. infantum*, *L. major*, and *L. tropica* in Turkey and Cyprus^14,15^; and *L. infantum* in Spain and Portugal^16^. *Leishmania (Viannia)* species is the predominant source of New World CL in Central and South America^17,18^.

According to the World Health Organization (WHO), approximately 600,000–1 million new cases of leishmaniasis are diagnosed worldwide annually^19^. Of these new cases, 70% of them are diagnosed in the Middle East region^19^. Saudi Arabia is considered the fourth country most endemic with CL in the Middle East region^20^. Various Saudi provinces are endemic to CL, including the Al-Ahsa oasis, Al Qassim, and Al-Madinah Al-Munawwarah^21^. In a previously published retrospective study, the incidence of CL in Al-Ahsa declined from 1956 confirmed cases in 2000 to 457 cases in 2010^22^.

Presumptive CL diagnosis can be established based on clinical presentation; however, establishing the definitive diagnosis of CL is relatively challenging, especially in endemic areas^23^. To date, microscopic identification of amastigotes in stained smears and promastigotes in tissue cultures are considered the gold standard diagnostic method^23^. Despite the high specificity of these techniques, they have limited sensitivity, are time-consuming, and cannot differentiate between *Leishmania* species^23^. Molecular techniques, such as polymerase chain reaction (PCR)-restriction fragment length polymorphism (PCR–RFLP), real-time PCR, and sequencing, are increasingly utilized for *Leishmania* speciation^24–30^. Multiple distinct genomic regions of *Leishmania* species have been evaluated using many different primer’s targets by PCR, such as kinetoplast DNA (kDNA), internal transcribed spacer one (ITS1) and ITS2, mini-exon, small subunit rRNA (ssu rRNA), and heat shock protein 70^24–30^.

Documenting the molecular profile of *Leishmania* species in Saudi Arabia can aid in providing effective treatment regimens for patients. Furthermore, exploring *Leishmania* transmission dynamics in vectors will help in designing high-quality preventative measures. Despite the prevalence and endemicity of the disease in Saudi Arabia, the genetic diversity of *Leishmania* species remains poorly studied^21,31–34^.

The current study aimed to identify *Leishmania* and speciate *Leishmania* molecularly from skin punch biopsy of clinically diagnosed CL cases from Al-Ahsa, Eastern Saudi Arabia.

## Methods

### Study area and study type

This was a cross-sectional diagnostic study conducted in Al-Ahsa City, Eastern Region, Saudi Arabia (coordinates, 25°2300″N 49°36′00″E) in collaboration with the Vector-Borne Diseases Prevention Center, Al-Ahsa.

### Ethical considerations

The study protocol was approved by the Institutional Review Board (IRB) Committee of the Deanship of Scientific Research at Imam Abdulrahman Bin Faisal University, and all methods were performed according to the relevant guidelines and regulations of the IRB Committee of Deanship of Scientific Research at Imam Abdulrahman Bin Faisal University (reference number: IRB-PGS-2020–01-427/IRB-PGS-2020–01-186) and Ministry of Health, Saudi Arabia IRB Committee (KFHH RCA #:08–25-2020). The purpose of the present study was comprehensively explained to all of the participants and the children’s parents/guardians, and the collection of specimens was completed after obtaining their informed consent.

### Sample collection

Skin biopsies were collected by punch biopsy (Kai Industries Co., Ltd., Japan) from patients clinically diagnosed with CL who were referred to the referral district leishmaniasis clinic at Al-Yahya Primary Health Care Center, Al-Ahsa, for the treatment of leishmaniasis.

Clinically suspected CL skin lesions were primarily diagnosed by the presence of ulcerated lesions or infiltrative erythematous nodules on exposed areas of the body of patients living in Al-Ahsa who had a history of exposure to sandfly bites. All patients diagnosed with CL who were referred to the leishmaniasis clinic during the study period were included in this study.

One punch biopsy was obtained from the borders of the lesion under sterile conditions. A total of 100 skin biopsies were performed between September 2020 and March 2021.

The related patients’ demographic and clinical data were collected using a designed questionnaire, including date of diagnosis, sex, age, nationality, place of residence, occupation, level of education, number of lesions, size, site, distribution and duration, family history of CL, treatment regimen, and response to treatment.

### Sample processing and microscopy

An impression smear was prepared by pressing the biopsy on the slide multiple times and then was stained with Giemsa stain. The slides were examined for amastigotes by 100 × oil-immersion light microscopy following practical guide for the diagnosis of leishmaniasis.^35,36^ Subsequently, the sample was stored at –20 °C for molecular analysis.

### Molecular assays

**Figure 1:**
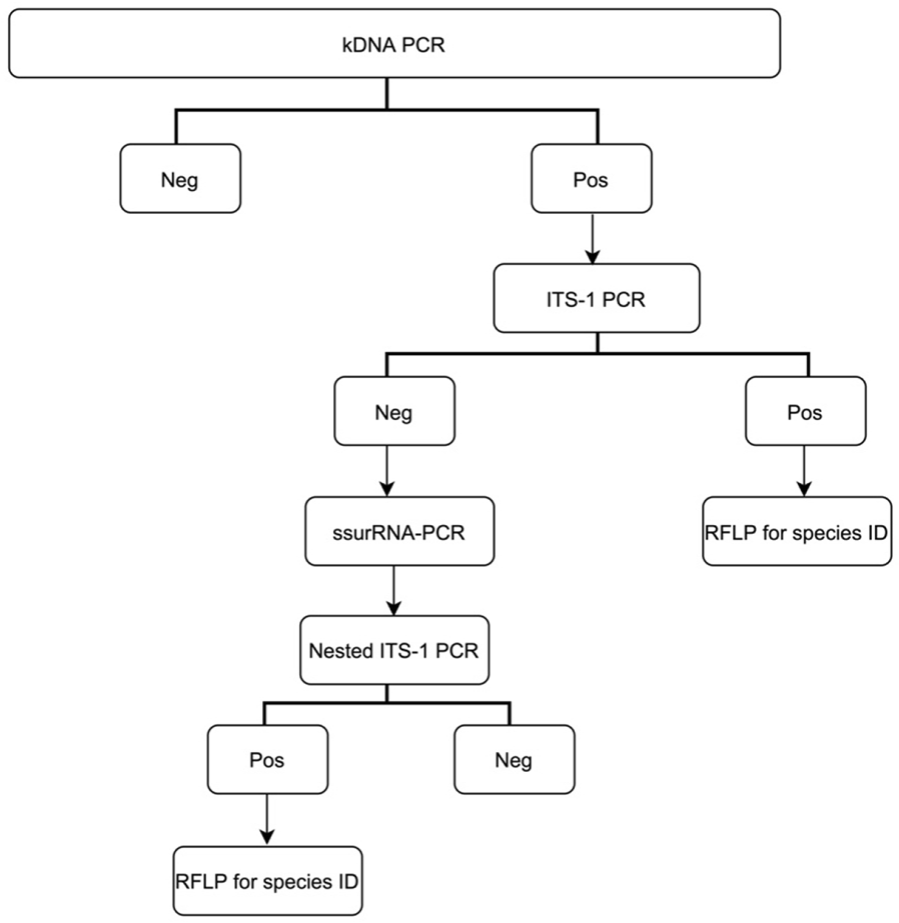
PCR-based Protocol for identification and speciation of *Leishmania* (Modified from Schönian et al., 2003 and El-Beshbishy et al., 2013)^29,32^.

Commercial genomic DNA extraction kits (QIAamp Fast DNA Tissue Kit, Qiagen, Germany) were used to extract the genomic DNA from skin specimens following the manufacturer’s guidelines. Extracted DNA was stored at 4 °C until processed. DNA extracted from samples was amplified for *Leishmania* DNA using three PCRs, a genus-specific kDNA PCR^32^ and two genus- and species-specific PCRs, the ITS1 PCR^29^ and ITS1 nested PCR (ITS1 nPCR)^29^. ITS1 nPCR was performed using two sets of two pairs of primers, the primers R221 and R332 targeting ssu rRNA and the nested primers LITS and L5.8S targeting ITS1. All samples were processed using a modified protocol of genus-specific primers targeting kDNA^32^ as a first-step screening tool, followed by the protocol published by Schönian et al.^29^ which uses two genus- and species-specific PCRs (Fig. 1).

A total of 25 µL was used for all PCR amplifications. Each reaction had 12.5 uL of Master mix, one uL of each primer, 7.5 uL RNA-free water, and three uL of sample’s DNA which was around 25 ng. *Leishmania major* MHOM/TM/82/Lev and *Leishmania tropica* MHOM/SU/80/K28 strains were used as positive control and RNA-free water were used as negative controls, respectively. PCR inhabitation control reactions were carried out to exclude false-negative results. All PCR reagents were purchased from Promega (USA). Primers were purchased from Macrogen (Seoul, South Korea). Table 1 shows the primers used and reaction cycling conditions applied for each PCR assay used in this study.

**Table 1:** PCR targets, primers, amplicon size and thermal cyclic conditions of the study PCR assays.

PCR target	Primer	Amplicon size	Thermal conditions	References
kDNA	13A:5′-GTGGGGGAGGGGCGTTCT 3′	120 BP	94 C (4 min), [94 C (30 s), 60 C (40 s), 72 C (1 min) for 35 cycles], 72 C (7 min)	^23^
	13B: 5′- ATTTTACACCAACCCCCAGTT-3′			
ITS1	LITSR: 5′- CTGGATCATTTTCCGATG-3′	320 BP	95 C (2 min), [95 C (30 s), 53 C (40 s),72 C (1 min) for 34 cycles], 72 C (7 min)	^37^
	L5.8S: 5′ TGATACCACTTATCGCACTT-3′			
ssu rRNA	R221: 5′- GGTTCCTTTCCTGATTTACG-3′	603 BP	94 C (3 min), [94 C (1 min), 56 C (1 min s),72 C (2 min) for 37cycles], 72 C (6 min)	^38^
	R332: 5′-GGCCGGTAAAGGCCGAATAG-3′			

All PCR amplified products were analyzed by electrophoresis on 1% agarose at 120 V in 1 × Tris–acetate-ethylenediaminetetraacetic acid buffer and were visualized under ultraviolet light.

### Genotyping of *Leishmania* species

*Leishmania* species were determined using ITS1 PCR/nPCR-RFLP and sequencing. All positive PCR products from direct ITS1 PCR and ITS1 nPCR were analyzed using restriction enzyme analysis for species identification.

Each PCR-amplified product (10 µL) was digested with 1 U *HaeIII* enzyme (MOLEQULE-ON, New Zealand) at 37 °C for 15 min, following the kit guidelines. The RFLP product was examined on MetaPhor (4%) agarose gel (Lonza, Switzerland) and visualized under ultraviolet light.

The ITS1 PCR/nPCR products were processed by the Macrogen Company (South Korea) for sequencing. Sequencing was conducted in both directions.

All obtained sequences were submitted to the GenBank database (accession numbers OK560721-OK560817).

All sequences were compared to the reference ITS1 sequences published in GenBank using BLAST (http://www.ncbi.nlm.nih.gov). DNA sequences were aligned and trimmed using the BioEdit software (version 7.2.5.0; http://www.mbio.ncsu.edu/BioEdit/bioedit.html). Neighbor-joining analysis was used to generate the phylogenetic tree using MEGAX software^39^. Bootstrap analyses with 1000 replicates were performed to assess the robustness of the constructed phylogenetic tree.

### Statistical analyses

Based upon previous published studies, ITS1 nPCR for negative cases by ITS1 PCR was used for accurate molecular diagnosis and was considered the reference standard diagnostic test^29,32,40–43^. The sample was considered positive if it was positive by direct ITS1 PCR or ITS1 nPCR. Data were analyzed using the Statistical Package for the Social Sciences (SPSS) software version 26 (SPSS Inc., Chicago, IL, USA). Descriptive statistics for categorical variables are presented as percentages and frequencies. Statistical significance was determined using the chi-squared tests, where the *P* value was less than 0.05. Specificity, positive predictive value (PPV), sensitivity, negative predictive value (NPV), accuracy, and kappa agreement were determined to evaluate the performance of each diagnostic method. Data are presented as tables, when appropriate.

### Ethical approval

The study protocol was approved by the IRB Committee of the Deanship of Scientific Research at Imam Abdulrahman Bin Faisal University (reference number: IRB-PGS-2020–01-427/IRB-PGS-2020–01-186) and Ministry of Health, Saudi Arabia IRB Committee (KFHH RCA #: 08–25-2020).

### Informed consent

The purpose of the present study was comprehensively explained to all of the participants and the children’s parents/guardians, and the collection of specimens was completed after obtaining their informed consent.

## Results

### Microscopic examination

**Figure 2:**
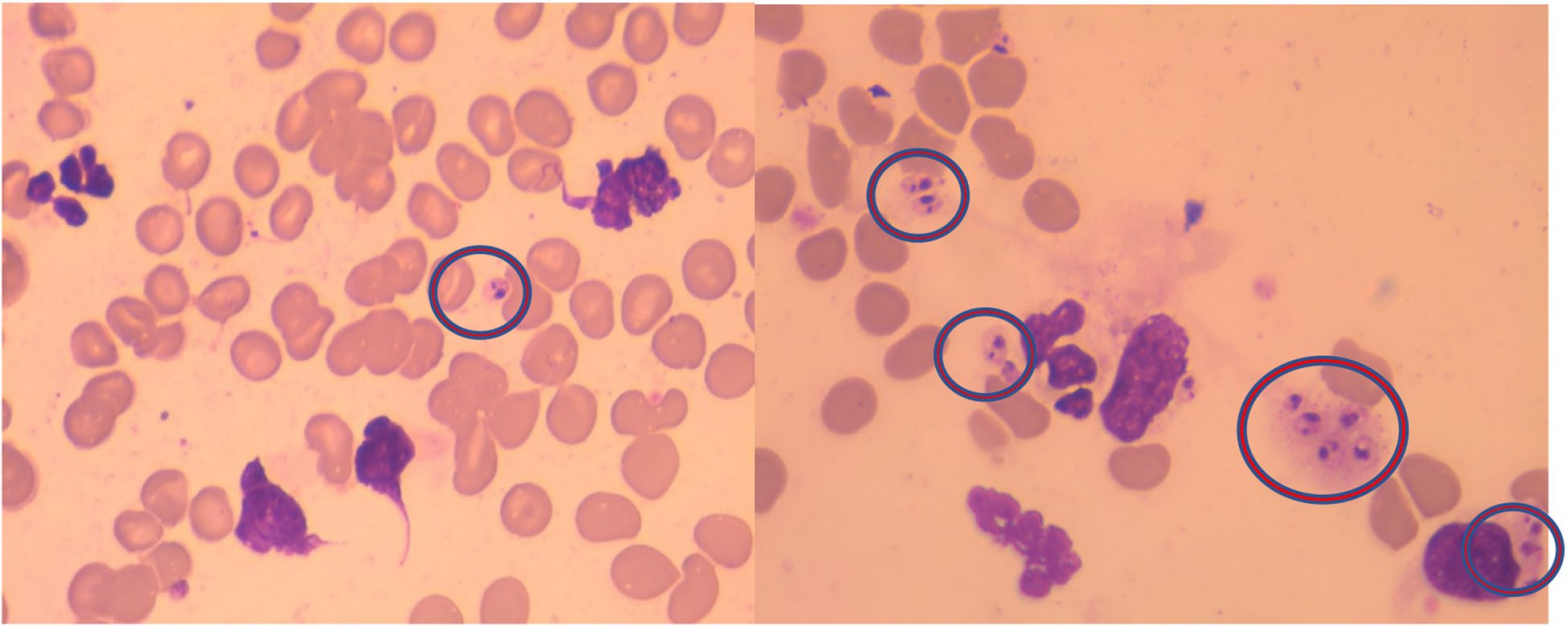
Geimsa stained impression skin smears showing *Leishmania species* amastigotes (Circled).

Out of the 100 stained impression smears from skin punch biopsies, 33% (33/100) were positive for *Leishmania* amastigotes (Fig. 2), whereas for 67% (67/100) of the smears, *Leishmania* amastigotes could not be observed.

Compared to the reference standard method, smear microscopy had perfect specificity (100%) and PPV (100%), but had poor sensitivity (31.6%) and NPV (2.9%). The results showed poor agreement with the reference standard (kappa = 0.18) (Tables 2, 3).

**Table 2:** Results of direct ITS1 PCR, kDNA PCR and microscopy of stained smear in detection of CL among all study individuals.

		Reference standard (ITS1 PCR/nPCR)			*P* value
		Positive	Negative	Total	
ITS1 PCR	Positive	89 (90.8%)	0 (0%)	89	< 0.0001
	Negative	9 (9.2%)	2 (100%)	11	
kDNA PCR	Positive	98 (100%)	2 (100.0%)	100	NA*
	Negative	0 (0.0%)	0 (0.0%)	0	
Giemsa microscopy	Positive	31 (31.6%)	2 (100%)	31	0.042
	Negative	67 (68.4%)	0 (0.0%)	67	
Total		98	2	100	

*NA: No statistics are computed because kDNA is a constant.

**Table 3:** Diagnostic performance and Kappa agreement of the study diagnostic tests.

	ITS1 PCR	kDNA PCR	Microscopy
Sensitivity	90.8% (CI 95%: 0.835–0.981)	NA*	31.6% (CI 95%: 0.199–0.434)
Specificity	100% (CI 95%: 1–1)	NA*	100% (CI 95%: 1–1)
PPV	100% (CI 95%: 1–1)	NA*	100% (CI 95%: 1–1)
NPV	18.2% (CI 95%: 0.109–0.473)	NA*	2.9% (CI 95%: -0.022–0.079)
Accuracy	91.6% (CI 95%: 0.838–0.982)	NA*	33% (CI 95%: 0.212–0.448)
Kappa**	0.28 (CI 95%: 0.109–0.458)	NA*	0.18 (CI 95%: -0.029–0.066)

*NA: No statistics are computed because kDNA is a constant.

**Key for Kappa:˂0 = Poor agreement, 0.01-0.20 = Slight agreement, 0.21-0.40 = Fair agreement, 0.41-0.60 = Moderate agreement, 0.61-0.8 = Substantial agreement, 0.81-1.00 = Almost perfect agreement

### Polymerase chain reaction assays

Using the reference standard, 98 of the 100 cases were considered true-positive CL cases, whereas two of the 100 cases were considered true-negative cases. Based on direct ITS1 PCR 89 of the 98 (90.8%, *P* < 0.001) cases were detected, it had a sensitivity of 90.8%, poor NPV (18.2%), and perfect specificity (100%) and PPV (100%). The level of kappa agreement between the direct ITS1 PCR and reference standard was fair (kappa = 0.28) (Tables 2, 3). kDNA PCR was positive in all sample reactions with two false-positive cases (Tables 2, 3).

### Identification of *Leishmania* species using restriction fragment length polymorphism analysis

**Figure 3:**
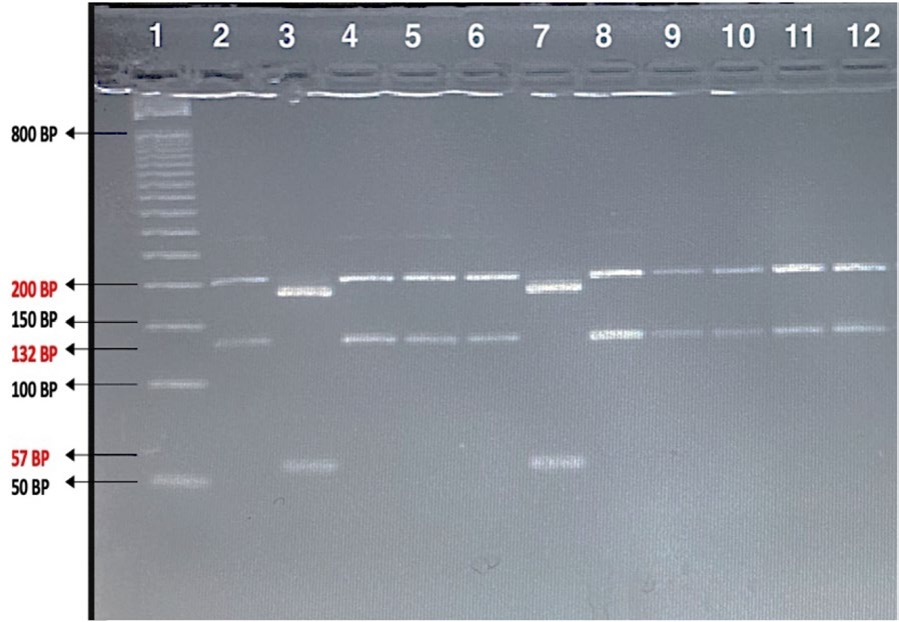
MetaPhor (4%) Agarose gel for *Leishmania* speciation isolated from clinical specimen of CL patients by analysis of restriction pattern of ITS1 PCR/nPCR products using restriction enzyme (*HaeIII*). Lane 1: 50 bp DNA ladder; lane 2: *L. major* MHOM/TM/82/ Lev positive control (203 and 132 bp); lane 3: *L. tropica* MHOM/SU/80/K28 positive control (200 and 57 bp); lanes 4–6 & 8–12: *L. major* samples; lane 7: *L. tropica* sample.

Identification of *Leishmania* species was performed by digesting PCR products for the 98 true-positive samples using *HeaIII* restriction enzyme to distinguish each sample’s RFLP pattern (Fig. 3). The predominant species was *L. major*; it was identified in 95 (96.9%) of the total patients’ sample. For the first time in Al-Ahsa, *L. tropica* was identified in three patients (3.1%) (Table 4).

**Table 4:** Results of genotyping using RFLP analysis.

*Leishmania species*	Number	Percentage
*L. major*	95	(96.9%)
*L. tropica*	3	(3.1%)

### Sequencing analysis

**Figure 4:**
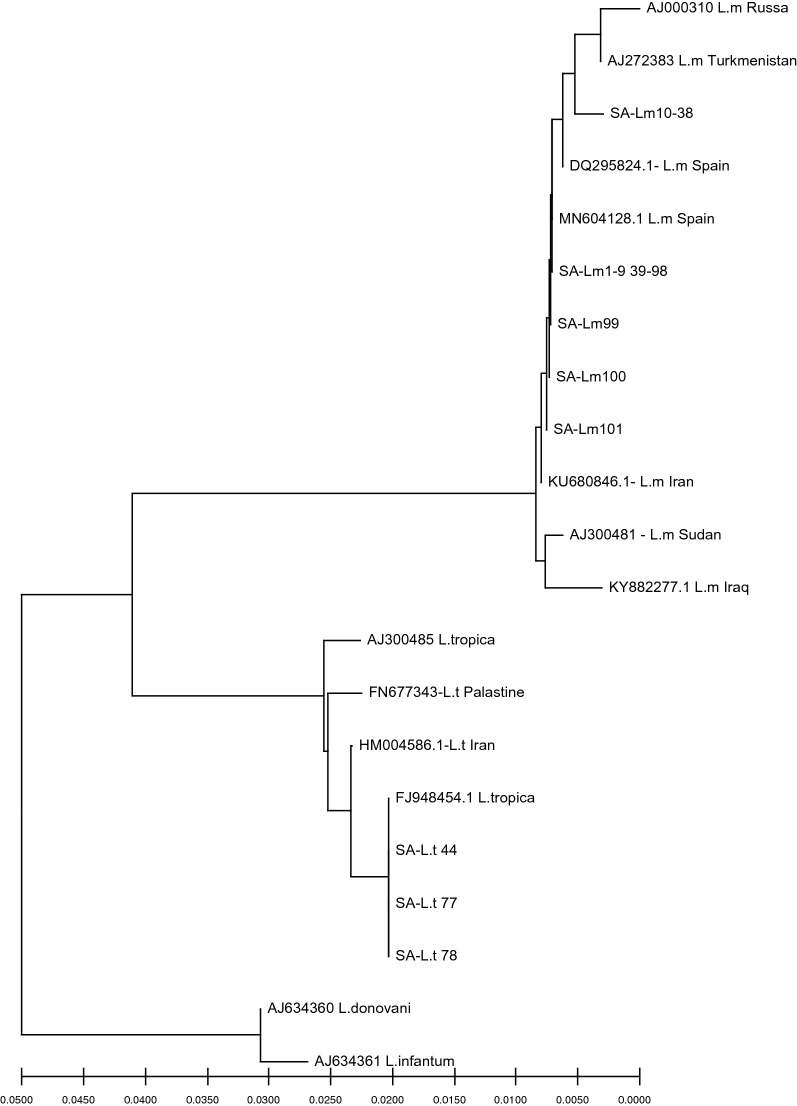
Dendrogram shows the Neighbor-joining phylogenetic tree of *L.major* (SA-L.m 1–9 and 39–98 in one cluster, 10–38 in one cluster, 99, 100 and 101) and *L.tropica* (SA-L.t 44, 77 and 78) study isolates relied on ITS1-5.8S rDNA gene sequences in comparison to reference strains (their accession number is located before their names). Similar sequences were clustered in the tree. Bootstrap analysis was relied on 1000 replicates. The distance scale at the bottom of the tree represents the number of differences between the sequences.

Sequencing analysis confirmed RFLP results with 100% similarity of the study DNA sequences with the sequences previously submitted to the GenBank, as shown in the constructed phylogenetic tree (Fig. 4).

### Sociodemographic and clinical data of study individuals and their association with the occurrence of cutaneous leishmaniasis and *Leishmania* species

Most patients were men (90%) and lived in rural areas (98%). More than half (61%) of the patients with CL were aged 15–44 years. There was no travel history for the participants in the last 6 months prior to the presentation.

**Figure 5:**
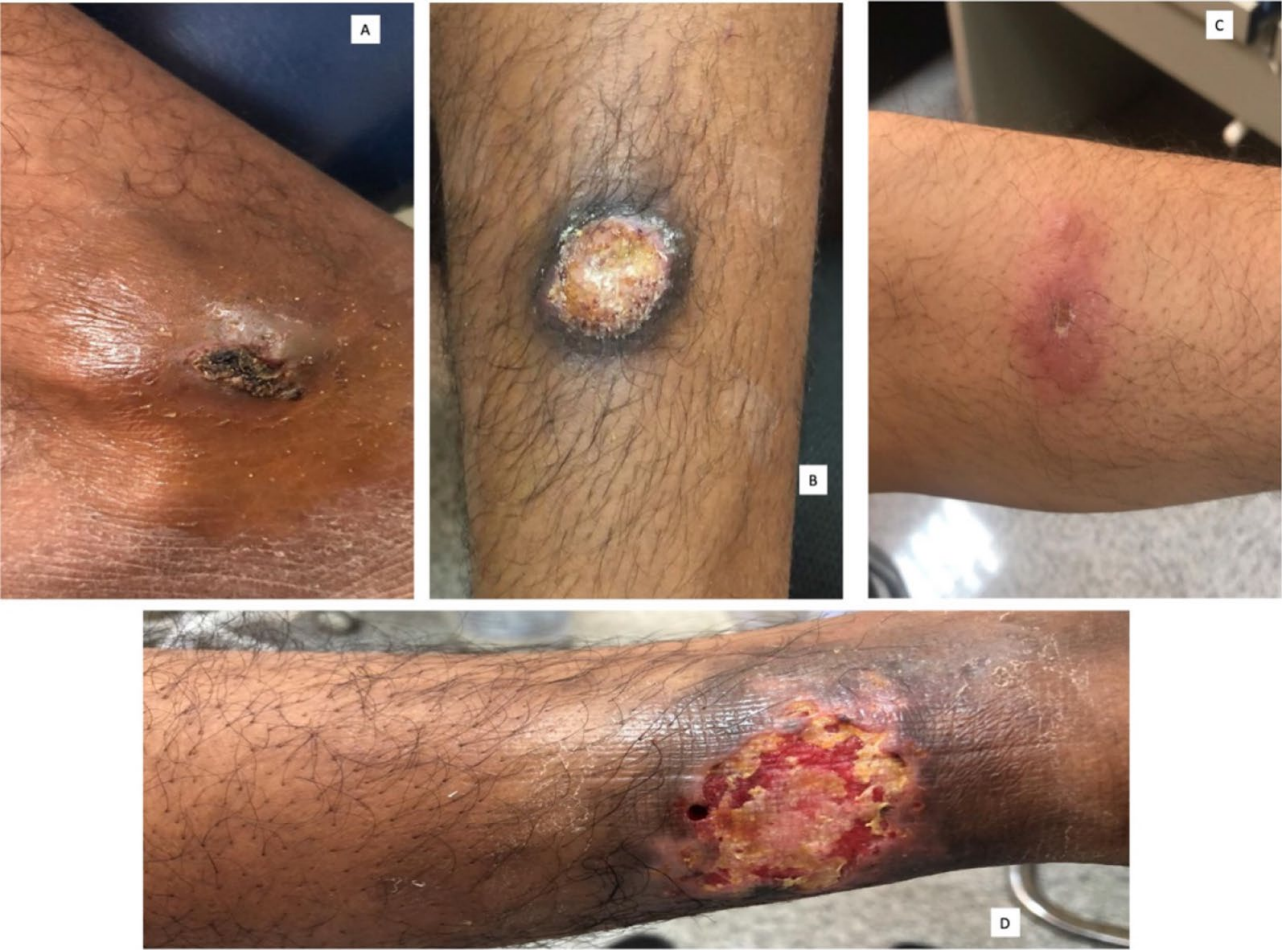
Different skin manifestations of CL patients. A: Right leg nodular lesion (1.5–2 cm) in a 43-year-old male laborer; B: Right leg ulcerative plaque lesion (4 cm) in a 37-year-old male farmer; C: Left forearm plaque lesion (0.5–1 cm) in a 48-year-old male truck driver; D: Left leg ulcerative lesion (5–5.5 cm) in a 31-year-old male driver.

Skin lesions in the patients with CL were distributed in different body areas, and 50% of the lesions were distributed in the legs. Most of the patients (76.5%) had skin lesions at one body site. The patient presented with various cutaneous lesions (Fig. 5). Ulcerative plaque lesions were the most common presentation (28%), followed by ulcerative lesion (19%), then nodular (17%), and plaque (13%) lesions.

**Figure 6:**
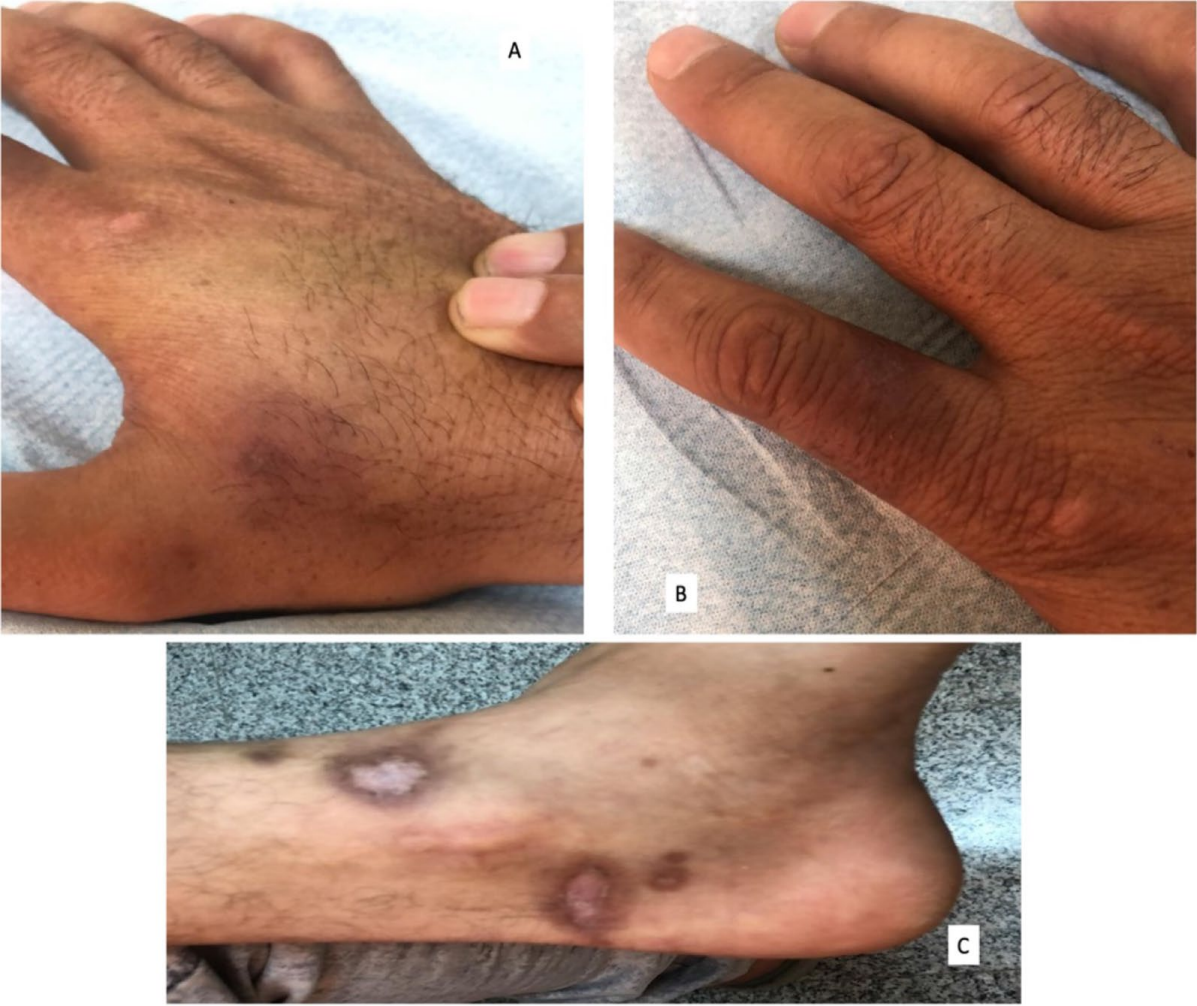
Resolution of the CL lesions. A-C: Post inflammatory hyperpigmentation and scaring in a different exposed body sites after a full course of treatment. (Once weekly 0.5 mL per lesion (50 mg) intralesional sodium stibogluconate for 6 weeks course).

The patients were treated according to the Saudi Ministry of Health CL treatment guidelines^44^. The choice of therapy was decided according to the clinical presentation, stage, number, and sites of the CL skin lesions. Most of the patients (94/98, 96%) were treated with sodium stibogluconate either intramuscularly or intra-lesionally. Only four (4%) patients were treated with oral fluconazole. Complete resolution of lesions after the entire course of treatment was achieved in 95 (96.9%) patients (Fig. 6). One patient required a second course of oral fluconazole for 4 weeks after the first course to achieve complete resolution. One patient had recurrence of the lesions after a full course of therapy (lesions appeared after 4 months of complete resolution).

The sociodemographic and clinical data of the patients were analyzed for their association with the molecular diagnosis of *Leishmania* and *Leishmania* species using ITS1 PCR/nPCR-RFLP (Tables 5 and 6). Among the studied patient characteristics, only sex, residence, and treatment outcome were associated with the occurrence of *Leishmania* with statistical significance (*P* < 0.05). Distribution of skin lesion(s) and treatment outcome were statistically significantly associated with the genotype of *Leishmania* (*P* < 0.05).

**Table 5:** Socio-demographic data of study individuals in association with *Leishmania* [ITS1 PCR/nPCR results] and *Leishmania* species [ITS1 PCR/nPCR-RFLP results].

		*Leishmania* and *Leishmania* species [ITS1 PCR/nPCR results] (n = 100)						
		Positive (RFLP results)				Negative	Total	*P* value
		*L.major*	*L.tropica*	*P* value	Total			
Sex	Male	86 (96.6)	3 (3.4%)	0.576	89 (98.9)	1 (1.1)	90	0.057
	Female	9 (100)	0 (0%)		9 (90)	1 (10)	10	
Nationality	Saudi	38 (97.4)	1 (2.6%)	0.816	39 (97.5)	1 (2.5)	40	0.773
	Non-Saudi	57 (96.6)	2 (3.4%)		59 (98.3)	1 (1.7)	60	
Residence	Rural	94 (96.9)	3 (3.1%)	0.858	97 (99)	1 (1)	98	**0.001**
	Urban	1 (100)	0 (0%)		1 (50)	1 (50)	2	
Employment	Employee	76 (97.4)	2 (2.6%)	0.573	78 (98.7)	1 (1.3)	79	0.309
	Unemployed	19 (95)	1 (5%)		20 (95)	1 (5)	21	
Level of education	Illiterate	17 (94.4)	1 (5.6%)	0.445	18 (100)	0 (0)	18	0.827
	Pre-school	7 (87.5)	1 (12.5%)		8 (100)	0 (0)	8	
	Primary School	18 (94.7)	1 (5.3%)		19 (95)	1 (5)	20	
	Intermediate School	15 (100)	0 (0%)		15 (100)	0 (0)	15	
	Secondary School	30 (100)	0 (0%)		30 (96.8)	1 (3.2)	31	
	Bachelor	8 (100)	0 (0%)		8 (100)	0 (0)	8	
Age group	1–4	2 (100)	0 (0%)	0.108	2 (100)	0 (0)	2	0.061
	5–9	3 (75)	1 (25%)		4 (80)	1 (20)	5	
	10–14	7 (100)	0 (0%)		7 (100)	0 (0)	7	
	15–44	58 (96.7)	2 (3.3%)		60 (98.4)	1 (1.6)	61	
	> 45	25 (100)	0 (0%)		25 (100)	0 (0)	25	
Total		95 (96.9)	3 (3.1)		98 (98)	2 (2)	100(100)	

Presented as number (percentage). P value is significant if > 0.05.

*Preschool: Participants still did not reach school age.

**Table 6:** Clinical data of the study individuals in association with *Leishmania* [ITS1 PCR/nPCR results] and *Leishmania* species [ITS1 PCR/nPCR-RFLP results].

		*Leishmania* and *Leishmania* species [ITS1 PCR/nPCR results] (n = 100)						
		Positive (RFLP results)				Negative	Total	*P* value
		*L. major*	*L. tropica*	***P* value**	**Total**			
Lesion sites	Single site	73 (97.3)	2 (2.7)	0.682	75 (97.4)	2 (2.6)	77	0.435
	Multiple sites	22 (95.7)	1 (4.3)		23 (100)	0 (0)	23	
Distribution of lesions	Legs	48 (98)	1 (2)	**0.038**	49 (98)	1 (2)	50	0.929
	Arms	25 (96.2)	1 (3.8)		26 (96.3)	1 (3.7)	27	
	Arms & Legs	13 (100)	0 (0)		13 (100)	0 (0)	13	
	Face & Extremities	2 (66.7)	1 (33.3)		3 (100)	0 (0)	3	
	Other*	7 (100)	0 (0)		7 (100)	0 (0)	7	
Number of lesions	1	40 (100)	0 (0)	0.118	40 (100)	2 (4.8)	42	0.589
	2–3	26 (89.7)	3 (10.3)		29 (100)	0 (0)	29	
	4–5	19 (100)	0 (0)		19 (100)	0 (0)	19	
	6–10	7 (100)	0 (0)		7 (100)	0 (0)	7	
	> 10	3 (100)	0 (0)		3 (100)	0 (0)	3	
Size of lesions (cm)	< 1	2 (100)	0 (0)	0.902	2 (100)	0 (0)	2	0.219
	1–2	21 (95.5)	1 (4.5)		22 (95.7)	1 (4.3)	23	
	3–5	61 (96.8)	2 (3.2)		63 (100)	0 (0)	63	
	> 5	11 (100)	0 (0)		11 (91.7)	1 (8.3)	12	
Clinical Outcome	Complete resolution after full course	93 (97.9)	2 (2.1)	**0.001**	95 (99)	1 (1)	96	**0.001**
	Resolution after repeated course of treatment	0 (0)	1 (100)		1 (100)	0 (0)	1	
	Recurrence after complete resolution	1 (100)	0 (0)		1 (100)	0 (0)	1	
	Not treated	1 (100)	0 (0)		1 (50)	1 (50)	2	
Family History	Yes	16 (88.9)	2 (11.1)	**0.028**	18 (94.7)	1 (5.3)	19	0.259
	No	79 (98.8)	1 (1.3)		80 (98.8)	1 (1.2)	81	
Total		95 (96.9)	3 (3.1)		98 (98)	2 (2)	100(100)	

*Other: Sites include abdomen, chest, shoulder, and buttock.

Significant values are in bold.

## Discussion

Molecular assays, such as PCR, are highly specific and sensitive for the diagnosis of infectious diseases, including CL. The molecular identification of *Leishmania* species was performed by following a modified protocol for genus-specific primers targeting kDNA as the first screening step^32^, followed by the use of two genus- and species-specific PCRs, ITS1 PCR and ITS1 nPCR, using the first ssu rRNA PCR (Fig. 1). This modification aimed to study the use of kDNA primer as a first-step screening tool to be implemented in routine diagnostic laboratories and thus overcome the possible false-negative results by Schönian et al.^29^. In our study, 98 of the 100 samples were considered true positive using our reference standard molecular method. Treatment was initiated by the clinical team if the sample was true positive.

The PCR of kDNA was positive in all samples, including two false-positive results. Clinical confirmation of false positivity was performed in the case without initiation of the CL treatment protocol. Both patients showed spontaneous resolution of the lesions after two weeks. kDNA PCR was able to detect as low as 10^–4^ parasites in patient samples^45^. Compared to other DNA targets, the kDNA target has abundant copy numbers (almost 10,000 copies) that are 50–250-fold higher than those of other PCR targets^23^. Based on our findings, the use of kDNA as a screening tool is discouraged as it may lead to false-positive results and is an extra step, whereas using ITS1 nPCR for negative cases by ITS1 PCR allows for accurate diagnosis and speciation of *Leishmania*. Other studies have suggested the use of kDNA as a screening tool with excellent performance^32,46^. Further comprehensive studies using larger sample sizes are required to assess kDNA PCR as a first-step screening tool. Based on direct ITS1-PCR, 89 of the 98 (90.8%, *P* < 0.001) cases were detected with 100% specificity and 90.8% sensitivity. Most published studies have reported similar results, with a sensitivity ranging from 63.5% to 100%^23,32,46–49^ and a specificity ranging from 93 to 100%^23,32,47–49^. The different methods used for sample collection in published studies could explain the wide range of sensitivities. Studies using punch biopsy or aspirate showed higher performance. Direct ITS1-PCR PPV was 100%, whereas the NPV was poor (18.2%). The poor NPV value compared to other published studies^23,32,48,49^ can be explained by our inclusion criteria, which only recruited patients referred to the regional CL clinic with a presumptive diagnosis of CL. Implementing ITS1 nPCR for negative samples using ITS1 PCR as a reference standard enhanced the sensitivity and overcame the false-negative results.

Sequencing analysis confirmed the ITS1 PCR/nPCR-RFLP results for *Leishmania* species identification. In the Al-Ahsa region, *L. major* was the predominant zoonotic species. Nevertheless, *L. tropica* was identified in three of the study participants. There was no travel history for any of the study individuals in the last six months prior to presentation. To the best of our knowledge, this is the first study to identify *L. tropica* in human CL cases in the Al-Ahsa region.

Previously, Al‐Salem et al.^33^ identified only *L. major* in clinical samples from the Al-Ahsa region. A similar species distribution was identified in other regions of Saudi Arabia. El-Beshbishy et al.^31,32^ found that *L. major* and *L. tropica* were the CL species present both in field-caught sandflies^31^ and in clinical samples^32^ in Al-Madinah Al-Munawwarah, western province of Saudi Arabia. The predominant *Leishmania* species was *L. major*. Rasheed et al.^50^ identified *L. major*, *L. tropica*, and a few cases of *L. infantum*/ *Leishmania donovani* in Qassim, central region of Saudi Arabia. In Asir and Jazan in southwestern Saudi Arabia, *L. tropica* is considered the predominant species^33^. Despite findings from all previous studies in Saudi Arabia, which exclusively reported the presence of the anthroponotic type of *L. tropica*, the zoonotic form of *L. tropica* was reported in Al-Ahsa in seven of the 175 samples collected from stray dogs by Alenezi et al.^51^. They only used two rounds of the less discriminative kDNA PCRs for genus and species identification to identify *L. tropica* in dogs, and they did not specify whether the samples had been collected from transported or local dogs.

In our study, the identification for the first time of *L. tropica* in human samples in the Al-Ahsa region can be explained by the migration of sandflies from other regions of Saudi Arabia during the COVID-19 pandemic lockdown that led to the cessation of vector-borne disease prevention programs and affected sandfly surveillance. The WHO and many experts have concluded that vector densities and health threats from vector-borne diseases can increase and even double as a result of restrictions in human mobility due to the COVID-19 lockdown, which may adversely affect the epidemiology of vector-borne diseases^52–55^.

More generally, the pattern of *Leishmania* species distribution in certain localities can easily change over time due to changes in the environment with rapid urbanization, vector movement, vector potency, vector control measures, animal transportation, and climate changes^56^.

Despite the poor performance of microscopic examination as a diagnostic tool, it is still considered the gold standard for diagnosing CL in clinically diagnosed patients in many laboratories worldwide^23^. Microscopy was limited by poor sensitivity, with missed diagnosis in 65 (66.3%) patients. Other studies have reported similar low sensitivity (37–62.6%) of microscopy to detect *Leishmania* amastigotes in stained skin smears^23,32,57–60^. The poor performance of this tool is explained by the need for a high number of viable parasites in the sample to be visualized, the extreme demand for an expert microscopist to read the smears, and the flawed methods of sample collection. Therefore, a reliable and cost-effective diagnostic tool for the diagnosis of CL is essential.

Multiple studies have addressed the relationship between the clinical and epidemiological characteristics of CL cases and the occurrence of *Leishmania* and *Leishmania* species^33,61–65^. In the current study, the area of residence of the study participants (*P* < 0.001) and the clinical outcome of treatment (*P* < 0.001) were significantly associated with PCR results, whereas family history (0.028), distribution of cutaneous lesions (0.038), and clinical outcome of treatment (*P* < 0.001) were significantly associated with ITS1-PCR RFLP results. In a previously published study in Saudi Arabia, multiple clinical factors, including lesion location, number, stage, and presence of satellite lesions, were significantly associated with species type^33^. El-Badry et al.^61^ reported a statistically significant association between the age group of affected individuals and the ITS1-PCR results in Libya. Other studies have reported a significant relationship between molecular characterization and different clinical and epidemiological factors, such as CD4 count^62^, presence of hyrax in the villages, cases of CL in neighboring houses^63^, history of chronic disease and duration of lesions^64^ and factors related to indoor dwelling environment^65^. These significant variables may also be predictors of the occurrence of *Leishmania* and *Leishmania* species in the region. The main limitation of our study is the lack of comprehensive clinical correlation between the species detected by the molecular techniques and the patients’ clinical presentation and demographics. Further studies are needed to confirm our results.

## Conclusion

To the best of our knowledge, this comprehensive molecular study fills a gap in the knowledge regarding the identification of the prevalent *Leishmania* species causing CL in the Al-Ahsa region. For the first time, our study detected human cases of anthroponotic *L. tropica* in Al-Ahsa. For accurate molecular diagnosis and speciation of *Leishmania*, we recommend using ITS1 nPCR for negative cases by ITS1 PCR*.* Further studies on vectors and animal reservoirs are essential to identify *Leishmania* transmission dynamics in the Al-Ahsa region. This crucial information can help guide the future planning of control methods to prevent the spread of CL in this region.

## Data Availability

The datasets generated and/or analyzed in the current study are available in the GenBank database (accession numbers: OK560721-OK560817).
